# The Psychological Symptoms of College Student in China during the Lockdown of COVID-19 Epidemic

**DOI:** 10.3390/healthcare9040447

**Published:** 2021-04-11

**Authors:** Yin Li, Linbo Qin, Yaobin Shi, Jun Han

**Affiliations:** College of Resources and Environment Engineering, Wuhan University of Science and Technology, Wuhan 430081, China; liyin@wust.edu.cn (Y.L.); qinlinbo@wust.edu.cn (L.Q.); shiyaobin@wust.edu.cn (Y.S.)

**Keywords:** psychological symptom, college student, COVID-19, lockdown

## Abstract

The COVID-19 epidemic has had a huge impact on the mental state of human beings due to its high infection and fatality rates in early 2020. In this paper, a cross-sectional online survey was designed to understand the mental state of college students in a university located in Wuhan city during the lockdown. Out of 1168 respondents, above 50% participants had obvious fear and anxiety symptoms; anxiety and fear were 61.64% and 58.39%, respectively. Conformity (49.49%), invulnerability (26.11%), insensitivity (21.49%) and rebelliousness (12.41%) symptoms also appeared. Meanwhile, it was revealed that the senior students experienced more anxiety than the freshmen. Moreover, it was found that the psychological symptoms (except for the insensitivity symptom) had no significant difference in gender, residence and annual household income after the one-way analysis of variance.

## 1. Introduction

In December 2019, COVID-19 first broke out in Wuhan city [1]. The virus could be spread by a person-to-person pattern including direct transmission, inhalation transmission and contact transmission. Afterwards, the virus quickly spread to China and the world [2]. Hence, COVID-19 was defined as a public health emergency of international concern on 30 January 2020 by the World Health Organization (WHO) and declared a pandemic on 12 March 2020 [3]. Up to 5 December 2020, there had been over 66 million reported confirmed cases of COVID-19 and 1.5 million deaths [4]. Wuhan city in the Hubei Province firstly implemented a Level 1 response to the public health emergency and a lockdown on 23 January 2020 due to the high fatality rate [1]. All public traffic was stopped and the movement of individuals was restricted. Most of the people, except for those involved in epidemic prevention and control, the police and few workers of necessary industries, were required to stay at home [5,6]. After Wuhan city, the government of all provinces in China implemented a Level 1 response to the public health emergency on 29 January 2020 [7]. Hence, all campuses of the universities in China were mandated to be closed in the spring of 2020 and the college students were required to stay at home and have their courses online to complete their academic study plan [8]. The long time of the lockdown caused people to get information, including a lot of fake news, from the media or the internet, which inevitably led to a stress response [9]. The transmission routes, origin and treatments of COVID-19 were not clearly understood at the early epidemic stage and the individual was only isolated in the home. Social interaction, physical activities and entertainment were prohibited and the normal living style was changed [10]. Meanwhile, surfing time increased and sleep and diets were irregular [11]. Thus, the long lockdown caused severe psychological symptoms such as anxiety, depression, insomnia and fear to the people isolated at home [12]. Qiu et al. carried out a survey and received 52,730 valid responses from 36 provinces as well as Hong Kong, Macau and Taiwan on 10 February 2020; they reported that 35% of the population in China experienced psychological distress during the COVID-19 epidemic [13]. A total of 1210 respondents from 194 cities in China took part in the survey within the first two weeks of the COVID-19 outbreak and the results of Wang et al. demonstrated that 28.8% of respondents had an anxious symptom of a moderate to severe level and 16.5% experienced moderate to severe depression symptoms [14]. Mazza et al. claimed that females exhibited a greater level of distress than males and had a higher level of anxiety. In addition, the respondents of the age of 18–30 and above 60 years were easier to be affected by distress than those with an age range of 30–60 [15]. College students are in the late adolescence stage with a high neurodevelopmental risk. Moreover, the supervision or attention from parents was significantly decreased [16] and college students were more vulnerable than the adults [12]. It was found that 7.7% of students were depressive, which was higher than that of the general population during the COVID-19 pandemic [17]. Anxiety, depression, sleep difficulties and stress were regarded as the main manifestations of the psychological symptoms in the disaster [18,19]. Wang et al. reported that the psychological symptoms for college students were moderate to severe anxiety (28.8%), depression (16.5%) and stress (8.1%) [20]. Fu et al. investigated the influence of variables such as sex, age, grade, place of residence and parent’s education level on the anxiety of college students and claimed that the anxiety level of the students in the rural regions was higher than that of the urban regions and that the female students experienced more anxiety than the male students due to their biology [21].

In this paper, a cross-sectional online survey was designed to acquire the mental state of college students in a university of science and technology located in Wuhan city during the lockdown and the impact of the residence region (urban or rural), grade, gender and annual household income on the psychological symptoms was assessed. The survey results will help the office of student affairs to understand the mental state of the students. Based on previous publications, it was hypothesized that:

**Hypothesis** **1.** *There is a significant difference of psychological symptoms with regard to the gender.*

**Hypothesis** **2.** *There is a significant difference of psychological symptoms with regard to the residence.*

**Hypothesis** **3.** *There is a significant difference of psychological symptoms with regard to the annual household income.*

## 2. Materials and Methods

The survey was designed according to a study conducted in a Chinese university [22], the Generalized Anxiety Disorder 7-Item Scale (GAD-7) [23] and the guiding principles of emergent psychological crisis intervention in COVID-19 [24]. Information such as age, gender, grade, health state, residence, annual household income and grade were collected using single item measures. Anxiety was obtained according to GAD-7 and Cronbach′s alpha coefficient was 0.901. Invulnerability, conformity, insensitivity, rebelliousness and bravado were measured by a Likert-type scale of 1–4; “1 = Never or very rare”, “2 = Sometimes”, “3 = Often” and “4 = Very often or always”. The risk level of the living region was decided according to the data from the National Health Commission of the People’s Republic of China (a high risk region ≥ 50 confirmed cases in two weeks; the moderate risk region had < 50 confirmed cases and had new confirmed cases in two weeks; the mild risk region had no new confirmed case in two weeks). The cross-sectional survey was carried out from 1 April to 1 June 2020. The Questionnaire Star (https://www.wjx.cn, accessed on 30 June 2020) was used as the platform of this survey. The participants were recruited from the Wuhan University of Science and Technology (WUST) and the website of the survey was shared in WeChat or a QQ group of the students of WUST. Before the survey, an electronic informed consent was signed online. The participants were informed that the survey was anonymous and they could reject the survey for any reason. The study was approved by the Ethics Committee of Wuhan University of Science and Technology (protocol code 20200407, 4 February 2021). All data were analyzed by SPSS software and a one-way analysis of variance was applied to assess the significance of each factor.

## 3. Results

### 3.1. Characteristics of Participants

A total of 1168 students completed the survey (1237 students were informed and the valid rate was 94.4%). The age of the participants was 17–25; the numbers of freshmen, sophomores, juniors, seniors and graduate students were 397, 328, 102, 68 and 273, respectively. Of the respondents, 34.93% were female and 65.07% were male. A total of 599 lived in an urban region and 569 lived in a rural region. The annual household incomes were above 0.8 million (0.17%), 0.4–0.8 million (1.71%), 0.2–0.4 million (11.39%) and below 0.2 million (86.73%). About 99.66% participants were healthy and 0.34% were exposed, and no suspicion or infected existed. The number of participants who lived in the low risk regions was 385 (32.96%); those in the middle risk regions and the high risk regions were 681 (58.30%) and 102 (8.73%), respectively. The demographic characteristics of participants is summarized in Table 1.

### 3.2. Psychological Symptoms of College Students during the Epidemic

Table 2 presents that most of the respondents had symptoms of fear and anxiety. A total of 58.39% participants experienced fear due to the high contagion level of COVID-19 and the mild, moderate and severe symptoms were 49.14%, 8.22% and 1.03%, respectively. At the same time, about 62.64% students had anxiety symptoms during the epidemic; moderate and severe anxiety were 0.02% and 6.51%, respectively. 

### 3.3. Distribution of Anxiety by the Grade of College Students

It can observe from Table 3 that the high grade students had higher levels of anxiety than that of the freshmen and the percentage of freshmen, sophomores, juniors, seniors and graduate students who had fear symptoms were 53.15%, 61.59%, 58.82%, 60.29% and 61.54%, respectively.

### 3.4. Analysis of the Significance of Factors

A one-way within subject analysis of variance (ANOVA) was applied to assess the significance of each factor such as the gender, residence and annual household income, as shown in Table 4, Table 5 and Table 6. Table 4 demonstrates that the F values of the ANOVA were below 3.0 and the *p* values were above 0.05. Hence, it could be said that there was no difference in regard to the residence. Thus, Hypothesis 1 was not supported. 

The impact of the risk level of the residence on the anxiety variation of participants is demonstrated in Table 5. It was found that 8.73% lived in a high risk level region; 58.30% and 32.96% were in the regions of medium and low risk levels. At the same time, the participants who lived in the medium and high risk level regions had greater anxiety than those of the low risk level regions. In addition, most of the participants appeared in the mild level of anxiety in this study.

Similar to the case of anxiety, the participants located in the safer regions experienced less fear, as shown in Table 6. The fear level of most of the participants was normal and mild. The safer areas had fewer restrictions and the participants could have more social interactions, physical activities and supplies, which was of benefit for good emotions.

According to Table 7, the F values of all psychological symptoms were below 3.0 and the *p* values were also above 0.05. Hence, it can be said that there was no obvious difference in regard to the gender so Hypothesis 2 was not supported. 

Table 8 demonstrates that there were no significant differences in invulnerability, conformity, fear, bravado, rebelliousness and anxiety in regard to the annual household income so Hypothesis 3 was not supported. However, the economical level of the family had an obvious influence on insensitivity during the epidemic period (F = 3.668, *p* = 0.033). The students with a medium annual household income had a high level of psychological symptoms such as invulnerability, conformity, fear, rebelliousness and insensitivity and the participants who had the highest annual household income had the highest level of anxiety.

## 4. Discussions

In this survey, over half of all college students surveyed had psychological symptoms of fear and anxiety during the epidemic of COVID-19. In the early epidemic stage, the origin, transmission routes and suitable medicines were unknown. A lot of negative information and rumors about the virus emerged in the media or on the internet and governments had no time to debunk them. At the same time, college students were isolated in the home and spent a lot of time browsing the internet; thus, a greater number of negative psychological symptoms appeared [25]. College students are in the later stage of adolescence and their mental states can be more easily affected by the information from the internet or media [26]. Thus, the college students surveyed suffered from fear, anxiety, invulnerability, conformity, bravado, rebelliousness and insensitivity symptoms. In this survey, 62.64% students had anxiety symptoms during the epidemic. Batra et al. assessed the psychological impact of COVID-19 among college students and found that the prevalence of anxiety in China was 25.5% compared with 58.7% in other regions [27]. The government strengthened the management of information released, the frequency of news release conferences increased and information or knowledge acquisition methods were also added. Thus, college students could know more information about COVID-19 and the cognition of COVID-19 was rebuilt. The level of the psychological symptoms appearing in college students gradually decreased [26].

In addition, the high grade students experienced more anxiety. The emotion variation was related to the worry of being infected, social support, income and academic delay [28]. The freshmen would not consider the graduation, employment and practice course [21], which would relieve part of the stress and anxiety [29]. Moreover, the higher grade students had greater academic pressure [21]. 

According to the results of Li et al., the risk level of the community had a linear relationship with the psychological symptoms of the residents (Severe ≥ 10,000 confirmed cases: Hubei province; Moderate = 1000–9999 confirmed cases: Guangdong, Henan, Hunan and Zhejiang provinces; Mild ≤ 1000 confirmed cases: all other provinces) [30]. Budimir et al. also thought that the risk level of the epidemic had a significant influence on mental health, e.g., depression, anxiety and insomnia [31]. In this survey, it was revealed that about 0.6% respondents had infected relatives, which had a negative effect on the emotional state of the students. However, Moghanibashi-Mansourieh claimed that belief was rebuilt after the relatives recovered from COVID-19 and the infected relatives had a positive influence on anxiety and stress reduction [32]. 

This survey revealed that there were no significant differences in gender, residence and annual household income and all hypotheses were not supported although there were significant differences of economy, infrastructure and policy of the central government between the rural and urban regions in China. In general, the economic situation in the rural regions was usually lower than in the urban region. At the same time, college students had to complete their study online and the internet in the rural regions was worse than in the urban regions [33]. Moreover, the urban regions had more hospitals and the patients could be promptly treated [28]. Food and medical supplies were also preferentially provided. However, rural regions usually have a low population density and the risk level was lower than that of urban regions. Hence, the limits of COVID-19 prevention in urban regions were stricter than those of the rural regions. Thus, there was no significant difference in the psychological symptoms with regard to the residence. 

Insensitivity symptoms seemed to have a significant difference in the annual household income. The participants with a 0.2–0.4 Chinese Yuan (RMB) million annual household income had the highest level of insensitivity symptoms due to the middle-class family having no financial pressures. Rossell et al. reported that people under economic stress experience more negative emotions [34]. At the same time, the parents of the middle-class families had more time to accompany the children. The participants who had the highest household income had the highest anxiety because they were worried about their business.

Although females were more sensitive and emotional to the environment due to biological factors and females experienced greater anxiety and fear in the disaster [35], the present study also discovered that both female and male college students had similar negative psychological symptoms during the epidemic of COVID-19. This conclusion was consistent with previous studies [21,28].

In general, half of the participants had negative psychological symptoms. Suitable intervening methods or strategies such as online psychological counseling and online mental health education courses as well as opportunities for talking with classmates, friends or teachers should be provided, which could be useful for building a positive attitude and effectively reducing the stress or psychological symptoms. Social support was also important in maintaining the psychology of college students. In addition, the psychological resilience of college students played an important role in safeguarding their mental health [12]. Shorter studying hours, a reduced workload, keeping enough sleep time and regular eating of healthy food were should be considered to improve the resilient mentality [36].

## 5. Limitation

This study had a few limitations, which limited the application of this finding in other epidemics. Firstly, the study had a limited response of 1168 and all respondents came from one university located in Wuhan city. The generalizability of this finding was insufficient. Secondly, this study was undertaken between April and June, which was the medium stage of COVID-19; the views could not represent the final view such as the influence of infected relatives on the psychological symptoms of students. A secondary survey was necessary and the dynamic variations of the psychological symptoms should be tracked. Thirdly, the respondents were not equally distributed with regard to gender, grade and residence. Lastly, all data from the survey were obtained by self-reporting. 

Although there were a few shortcomings, this study gave the psychological symptoms of college students in the COVID-19 epidemic, which was useful for choosing a suitable method of psychological intervention.

## 6. Future Direction

This survey investigated the dependence of psychological symptoms such as fear, anxiety, conformity, invulnerability, insensitivity and rebelliousness on the gender, residence and annual household income during the epidemic of COVID-19. A longitudinal study of the psychological symptoms during and after the epidemic is necessary and the severity of psychological symptoms can be understood. In addition, the relationship between psychological symptoms and academic study also should be considered. Lastly, it is also worth considering the influence of the suggested intervention methods on psychological symptoms.

## 7. Conclusions

In general, all members of society have had a huge stress due to the high fatality rate of COVID-19. Especially for relatives or friends who were infected by or exposed to COVID-19, stress levels sharply increased. Thus, the body shows stress responses such as the variation of emotions, biology, behavior and cognition. In this study, it was revealed that half of the participants had obvious psychological symptoms during the epidemic of COVID-19. The psychological symptoms of the college students were anxiety (61.64%), fear (58.39%), conformity (49.49%), invulnerability (26.11%), insensitivity (21.49%) and rebelliousness (12.41%). In addition, about 6.16% students had insomnia and 2.83% had the symptoms of in-appetite.

Moreover, the results of the survey also presented that psychological symptoms had no significant difference with regard to the gender, residence and annual household income while the financial level of the family had an obvious influence on insensitivity symptoms during the epidemic period. The survey also revealed that the senior students experienced more fear and anxiety than the freshmen due to the worry about the academic study delay and practice courses, graduation and employment.

## Figures and Tables

**Table 1:** Demographic characteristics of participants (*n* = 1168).

Characteristics		Frequency
Gender	Male	408 (34.93%)
	Female	760 (65.07%)
Grade	Freshmen	397 (33.99%)
	Sophomore	328 (28.08%)
	Junior	102 (8.73%)
	Senior	68 (5.82%)
	Graduate student	273 (23.37%)
Residence	Rural	569 (48.72%)
	Urban	599 (51.28%)
Annual household income (RMB million)	>0.8	2 (0.17%)
	0.4–0.8	20 (1.71%)
	0.2–0.4	133 (11.39%)
	<0.2	1013 (86.73%)
Health state	Regular	1164 (99.66%)
	Exposed	4 (0.34%)
	Suspicion	0 (0.00%)
	Infected	0 (0.00%)
Risk level of living region	Low	385 (32.96%)
	Middle	681 (58.30%)
	High	102 (8.73%)

**Table 2:** Psychological symptoms of college students during the COVID-19 epidemic.

Psychological Symptoms	Normal	Mild	Moderate	Severe
Fear	41.61%	49.14%	8.22%	1.03%
Anxiety	38.36%	54.02%	0.00%	6.51%
Invulnerability	73.89%	22.86%	2.23%	1.03%
Conformity	50.51%	40.5%	8.3%	0.68%
Bravado	88.01%	11.04%	0.77%	0.17%
Rebelliousness	87.59%	10.36%	1.71%	0.34%
Insensitivity	78.51%	18.84%	2.05%	0.6%

**Table 3:** Distribution of anxiety by the grade.

Level of Anxiety	Freshman	Sophomore	Junior	Senior	Graduate	Total Number
Normal	46.85%	38.41%	41.18%	39.71%	38.46%	486
Mild	44.33%	50.61%	51.96%	52.94%	52.38%	574
Moderate	7.56%	9.76%	4.90%	5.88%	9.16%	96
Severe	1.26%	1.22%	1.96%	1.47%	0.00%	12

**Table 4:** The difference in psychological symptoms across the residences.

Variance	Residence (Mean ± SD)		F	*p*
	Urban (*n* = 599)	Rural (*n* = 569)		
Invulnerability	1.31 ± 0.58	1.30 ± 0.54	0.167	0.683
Conformity	1.58 ± 0.67	1.60 ± 0.67	0.146	0.703
Fear	1.68 ± 0.68	1.70 ± 0.65	0.218	0.641
Bravado	1.15 ± 0.41	1.11 ± 0.33	2.728	0.099
Rebelliousness	1.15 ± 0.46	1.14 ± 0.39	0.098	0.754
Insensitivity	1.26 ± 0.53	1.24 ± 0.49	0.436	0.509
Anxiety	2.32 ± 0.65	2.33 ± 0.64	0.071	0.790

F, test statistic; *p*, probability value.

**Table 5:** The impact of the risk level of the residence on anxiety.

Risk Level of Residence	Normal	Mild and Moderate	Severe	Total
High	25 (24.51%)	64 (62.75%)	13 (12.75%)	102 (8.73%)
Medium	255 (37.44%)	381 (55.95%)	45 (8.29%)	681 (58.30%)
Low	168 (43.64%)	186 (48.31%)	31 (8.05%)	385 (32.96%)

**Table 6:** The impact of the risk level of the residence on fear.

Risk Level of Residence	Normal	Mild	Moderate	Severe	Total
High	32 (31.37%)	51 (50.00%)	16 (15.69%)	3 (2.94%)	102 (8.73%)
Medium	253 (37.15%)	366 (53.74%)	56 (8.22%)	6 (0.88%)	681 (58.30%)
Low	201 (52.21%)	157 (40.78%)	24 (6.23%)	3 (0.78%)	385 (32.96%)

**Table 7:** The difference in psychological symptoms across the genders.

Variance	Gender (Mean ± SD)		F	*p*
	Male(*n* = 760)	Female (*n* = 408)		
Invulnerability	1.40 ± 0.50	1.20 ± 0.41	2.400	0.128
Conformity	1.60 ± 0.75	1.63 ± 0.56	0.032	0.858
Fear	1.60 ± 0.60	1.77 ± 0.73	0.722	0.400
Bravado	1.15 ± 0.49	1.03 ± 0.18	1.421	0.239
Rebelliousness	1.10 ± 0.31	1.03 ± 0.18	0.925	0.341
Insensitivity	1.25 ± 0.44	1.23 ± 0.50	0.014	0.905
Anxiety	1.95 ± 0.60	2.17 ± 0.70	1.281	0.263

F, test statistic; *p*, probability value.

**Table 8:** The difference in psychological symptoms across the annual household income.

Variance	Annual Household Income (RMB Million) (Mean ± SD)				
	<0.2 (*n* = 1013)	0.2–0.4 (*n* = 133)	>0.4 (*n* = 22)	F	*p*
Invulnerability	1.26 ± 0.44	1.38 ± 0.52	1.33 ± 0.58	0.241	0.787
Conformity	1.64 ± 0.63	1.75 ± 0.71	1.00 ± 0.00	1.661	0.201
Fear	1.72 ± 0.69	1.75 ± 0.71	1.33 ± 0.58	0.464	0.631
Bravado	1.08 ± 0.35	1.13 ± 0.35	1.00 ± 0.00	0.149	0.862
Rebelliousness	1.05 ± 0.22	1.13 ± 0.35	1.00 ± 0.00	0.403	0.670
Insensitivity	1.18 ± 0.39	1.63 ± 0.74	1.00 ± 0.00	3.668	0.033 *
Anxiety	2.08 ± 0.70	1.88 ± 0.35	2.67 ± 0.58	1.584	0.216

F, test statistic; *p*, probability value; * *p* < 0.05.

## Data Availability

The data presented in this study are available on request from the corresponding author.
